# Sports participation and physical health outcomes: the mediating roles of educational attainment and health-related behaviors

**DOI:** 10.3389/fpubh.2026.1912038

**Published:** 2026-09-21

**Authors:** Lei Zhang, Dongchen Qiao

**Affiliations:** 1 School of Physical Education, Xinyang Normal University, Xinyang, China; 2 Tangshan Maritime Institute, Tangshan, China

**Keywords:** educational attainment, health-related behaviors, physical health outcomes, public health, sports participation

## Abstract

Participation in sports has been associated with better physical health, but the educational and behavioral pathways through which this association operates have not been sufficiently investigated in public health research. Therefore, this study examined an integrated public health model from sports participation to physical health outcomes and the mediating effects of educational attainment and health-related behaviors. A quantitative cross-sectional survey design was used to investigate adults aged 18 years and older who had participated in organized or informal sporting activities. Data collection was conducted using a 20-item questionnaire developed by the researchers using adapted items from established validated instruments. The questionnaire assessed four constructs: Sports Participation, Educational Attainment, Health-Related Behaviors, and Physical Health Outcomes, with five items used to measure each construct. Of the 700 questionnaires administered, 586 were returned, of which 520 complete and eligible responses were retained for analysis. The data were examined using descriptive statistics, Pearson correlation, regression procedures, and bootstrap mediation testing based on 5,000 resamples. Sports participation showed significant positive associations with physical health outcomes (*β* = 0.41, *p* < 0.001) and educational attainment (*β* = 0.55, *p* < 0.001), whereas educational attainment also demonstrated a significant association with physical health outcomes (*β* = 0.36, *p* < 0.001). A statistically significant indirect effect was observed through educational attainment [indirect effect = 0.198, 95% CI (0.132, 0.271)] as well as through health-related behavioral patterns [indirect effect = 0.224, 95% CI (0.151, 0.306)]. The overall model explained 58% of the variance in physical health outcomes (*R*^2^ = 0.58). The study contributes by integrating educational and behavioral pathways within a unified sports health framework, with implications for integrated public health promotion, and coordinated interventions across educational, community, and health policy settings in practice.

## Introduction

Sports participation encompasses involvement in organized and informal sports, competitive activities, recreational exercise, and school- or community-based athletic programs (1). From a public health perspective, participation in sports represents more than the accumulation of physical activity (2). It constitutes a behavioral and social context in which regular movement, personal discipline, social interaction, and lifestyle routines may develop simultaneously (3). Consequently, engagement in sports may influence physical health through multiple interacting pathways instead of being explained solely by physical activity participation (4). Physical health represents the general state and performance of the body, encompassing fitness level, energy, functional ability, body-weight condition, and the existence or absence of illness or physical restrictions (5). In the present study, physical health outcomes represent these observable or perceived dimensions of bodily functioning and general physical well-being (6). Consistent involvement in sports may contribute to these outcomes by promoting greater engagement in physical activity while spending less time in sedentary pursuits (7). Health-related behaviors refer to everyday actions and lifestyle practices that can either protect or impair physical health (8). In the present study, the behaviors considered here comprise routine physical activity, balanced dietary intake, sufficient sleep and recovery, reduced sedentary time, avoidance of harmful habits such as smoking, and appropriate use of preventive health measures (9). These behaviors are closely interconnected because adequate sleep and nutrition can support recovery and physical functioning, regular activity can reduce sedentary exposure, and preventive behaviors can reduce vulnerability to avoidable health problems (10). Participation in sports may encourage greater attention to these practices because physical performance and recovery are influenced by nutrition, sleep, activity patterns, and other lifestyle choices (11). Consequently, health-related behaviors are conceptualized in this study as a proximal behavioral pathway through which involvement in sports may be linked with subsequent physical health outcomes (12).

Individuals who participate regularly in sports may become more attentive to nutrition, sleep, recovery, sedentary behavior, avoidance of harmful practices such as smoking, and preventive health behaviors because these practices can influence both sporting performance and general well-being (13).

Participation in sports may support educational development by promoting self-discipline, motivation, teamwork, time-management skills, goal-directed behavior, and social development (14). These behavioral and social competencies may contribute to greater educational engagement and academic performance among students and young adults (15). At the same time, the sports education relationship should not be regarded as necessarily unidirectional (16). People with more advanced educational attainment may possess broader knowledge about health, stronger socioeconomic advantages, increased awareness regarding the benefits of physical activity, and improved access to sports-related opportunities, factors that may further encourage their involvement in sports (17). Health-related behaviors are also theoretically connected with the other variables in the proposed model (18). Greater involvement in sports may encourage more regular physical activity, greater attention to nutrition and sleep, reduced sedentary behavior, and avoidance of harmful lifestyle practices (19). Educational attainment may further support healthier behaviors through improved health literacy, greater awareness of health risks, and better access to health-promoting resources (20). In turn, healthier behavioral patterns are associated with better physical functioning and general physical health (21). Therefore, health-related behaviors are theorised as a proximate mechanism linking participation in sports activities to physical health outcomes. Education may offer more general cognitive and socio-economic resources that facilitate healthier lifestyle choices (12). Educational attainment is also linked to better employment opportunities, better access to health-promoting resources, better health awareness, and better health decision making, making it a vital social determinant of health (22).

Thus, a reciprocal association between education and sports participation is theoretically plausible (23). The paper does not attempt to demonstrate that participation in sport has a causal effect on later educational attainment. Instead, we define sports participation as the focal predictor and educational attainment as an intermediate explanatory variable for testing the proposed conceptual model (24). This proposed direction represents the hypothesised mechanism by which participation-derived experiences, including discipline, engagement, and goal-directed actions, contribute to educational development, which in turn relates to physical health outcomes (25). Because the study uses a cross-sectional design, this relationship should be viewed as a theory-based predictive pathway rather than as evidence of a temporal causal effect (26).

Considering educational attainment together with health-related behaviors offers a more comprehensive account of the relationship between sports involvement and health (27). Educational attainment reflects a broader social and cognitive pathway connected with health literacy, available resources, and decision-making capacity, while health-related behaviors represent a closer lifestyle pathway involving everyday practices including nutrition, sleep patterns, physical activity, sedentary habits, and the avoidance of harmful practices (28). Sports involvement may consequently relate to physical health through both a direct route and two separate indirect mechanisms involving educational and behavioral pathways (29). Considering these mechanisms simultaneously provides a more comprehensive public health framework than examining sports health, sports education, or education health relationships independently (30).

Although previous research has documented associations between sports participation and health and between education and health, these relationships have frequently been examined separately (31). Less attention has been given to an integrated framework that evaluates educational attainment and health-related behaviors concurrently as explanatory mechanisms linking sports participation to physical health outcomes (32). This gap restricts the understanding of whether health benefits of sports participation are mainly due to direct physical involvement or if educational and behavioral mechanisms are also involved in the association (32).

This study therefore aims to assess an integrated model evaluating the association linking sports involvement and physical health outcomes, with educational attainment and health-related behaviors considered as mediating factors. More specifically, the present investigation evaluates the relationships among sports participation and physical health outcomes, educational achievement and health-related behavioral practices, the relationships linking educational attainment and health-related behavioral patterns with physical health outcomes, the mediating role of educational attainment and health-related behaviors in the association between sports participation and physical health outcomes, andthe indirect pathways through educational attainment and health-related behaviors linking sports involvement with physical health outcomes. The study is to examine these direct and indirect pathways to help elucidate the educational and behavioral mechanisms that may link sports participation to physical health.

### Conceptual framework and hypotheses

In the conceptual framework, the main predictor variable is sports participation, the main dependent outcome is physical health outcomes, and two theoretical mediating pathways are educational attainment and health-related behaviors. The framework assumes that participation in sport is embedded in wider educational, behavioral and social processes which may be relevant to physical health. Thus, the proposed model goes beyond a mere sports–health association to explore mechanisms that may statistically account for part of the relationship.

Figure 1 summarises the conceptual model proposed. The focal predictor is defined as sports participation and the outcome is physical health outcomes. Two parallel mediating mechanisms are labelled as educational attainment and health-related behaviors. The model therefore tests for a direct path between participation in sport and physical health outcomes, and two indirect paths: participation in sport → educational attainment → physical health outcomes and participation in sport → health-related behaviors → physical health outcomes. Because the present study uses a cross-sectional design, these pathways should be interpreted as theory-based predictive and mediating associations rather than as evidence of temporal causation. The first pathway considers the direct predictive link of sports participation with physical health outcomes (34).

**Figure 1:**
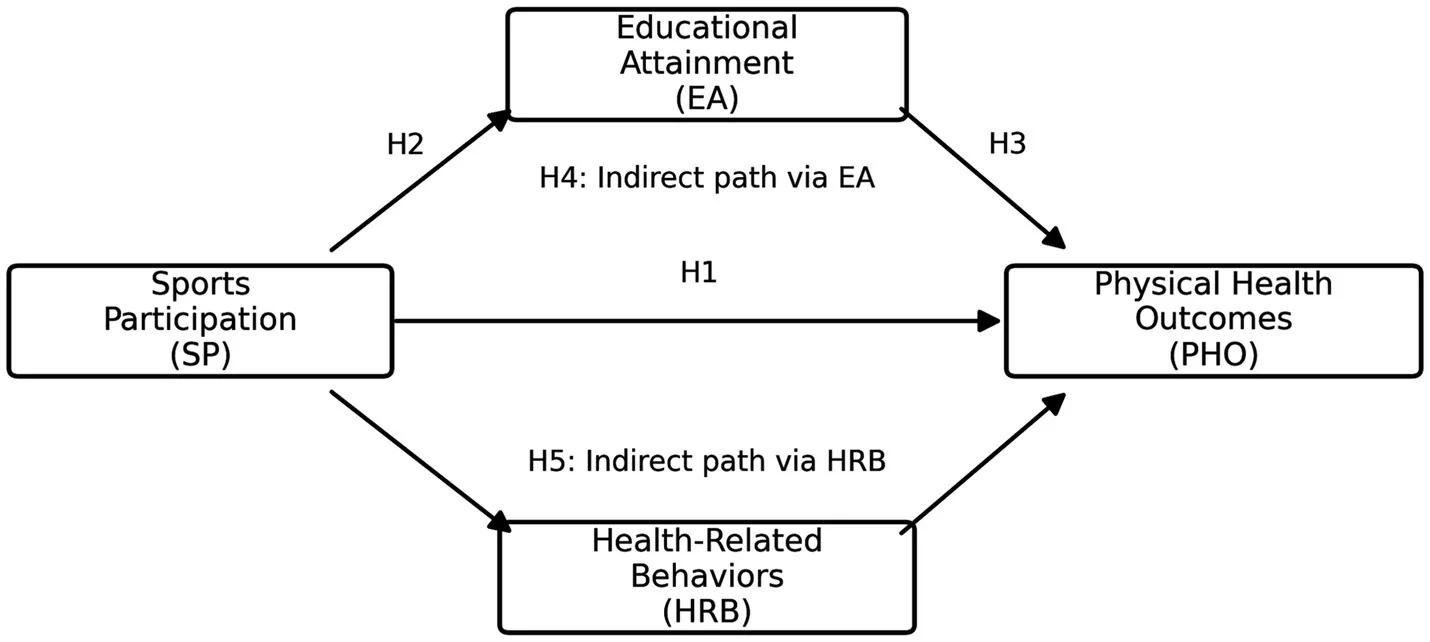
Proposed conceptual framework.

*H1*: Sports participation predicts positive physical health outcomes.

Participation in sports usually includes regular physical activity and less sedentary time and may promote physical functioning, fitness, energy and overall health (33). Regular physical activity can contribute to healthier body weight, whereas inactivity and prolonged sedentary behaviors are linked to higher risks of obesity, metabolic abnormalities, cardiovascular disease, and other chronic conditions (34). In the present framework, all these physiological benefits are taken together as the direct physical pathway from sport participation to physical health and not as separate entities (35).

*H2*: Sports participation positively predicts educational attainment.

The second pathway relates to educational attainment. Participation in organized sports can foster self-discipline, self-confidence, motivation, teamwork, time management, persistence, and goal-oriented behavior, all of which can contribute to educational engagement and academic development (36). In an educational context, sports participation can also strengthen students’ connection to schools or universities, encourage regular attendance and provide structured opportunities for social interaction and engagement with the institution (37).

Nevertheless, the relationship between sports participation and education may be reciprocal, because individuals with higher educational and socioeconomic resources may also have greater access to sporting opportunities (38). Thus, the directional pathway proposed in the current model should be understood as a theoretical predictive association rather than confirmation that sports participation directly leads to greater educational attainment (41).

*H3*: Education level is positively associated with physical health.

The third pathway examines the association between educational attainment and physical health outcomes (39). Higher levels of education may have positive effects on physical health outcomes through higher levels of health literacy, better understanding of health information, more informed health-related decision making, better employment and socioeconomic opportunities, and better access to healthcare and health-promoting resources (20). Education therefore represents an important social factor influencing health and provides a theoretical basis for hypothesising a positive association with physical health outcomes (40).

*H4*: Educational attainment mediates the association between participation in sports and physical health outcomes.

A fourth pathway links together the above relationships by implicating educational attainment as a potential mediating mechanism (41). Participation in sports can contribute to educational development through factors such as discipline, motivation, engagement with institutions, and other growth experiences. Conversely, greater educational attainment may enhance health literacy, socioeconomic opportunities, and access to resources that support better health (14). Thus, part of the link between sports participation and physical health outcomes may be statistically accounted for by educational attainment (45).

*H5*: Health-related behaviors mediate the association between sports participation and physical health outcomes.

The fifth pathway involves the mediating role of health-related behaviors. Regular participation in sports may be associated with a broader lifestyle of health-oriented behaviors. Adequate diet, sleep, recovery, regular activity, limited sedentary behavior and lack of harmful behaviors support both sports participation and health (42). These behaviors are directly related to physical health andmay help account for the association linking sports participation with physical health outcomes. Within this framework, health-related behaviors are the more proximal behavioral mechanism that complements the more distal educational pathway (12).

## Methodology

### Study design

This investigation adopted a quantitative correlational survey using a cross-sectional design. The quantitative approach was adopted to determine the relationships among Sports Participation, Educational Attainment, Health-Related Behaviors and Physical Health Outcomes using numerical measures. Data from participants were collected using cross-sectional approach within one period of time. Correlational analysis was used to assess the magnitude and direction of relationships among study variables. Then, regression and mediation analyses were conducted to test the theoretically specifieddirect and indirect predictive paths hypothesised in the model. Since the data were collected at one time, the observed relationships were viewed as statistical associations and predictive patterns, rather than evidence of longitudinal causation.

### Study group and participants

The target population consisted of adults aged 18 years and above who had participated in sports through school, college, university or community-based programs, recreational activities or informal physical exercise. Convenience sampling was used with stratified outreach procedures to ensure diversity across gender, age groups, educational backgrounds, residential characteristics and socioeconomic categories.

Exclusion criteria were age <18 years, no previous participation in organized or informal sport, and not meeting the pre-determined eligibility criteria for the study. During data screening, responses were excluded if questionnaires indicated a large amount of missing data, repeated submissions, irregular response patterns, or very short completion times that indicated a lack of engagement with the questionnaire.

The sample size was calculated according to the planned multivariable statistical analyses and the need to maintain a sufficient number of valid responses after screening. To cover potential non-response and unusable questionnaires, 700 questionnaires were distributed through educational institutions, community networks, sports clubs and online platforms. Among these, 586 were returned and 520 responses met the eligibility and data-quality criteria and were retained for the final analysis. The final sample was deemed adequate for conducting correlational, regression, exploratory factor, and mediation analyses.

The last group of subjects was composed of 52% males and 48% females. In relation to educational background, 22% of participants had completed secondary-level education, 58% were studying at or had completed undergraduate education, and 20% possessed postgraduate qualifications. Regarding residence, 61% of respondents belonged to urban areas, while 39% were from rural locations.

### Measures and data collection

Data were collected using a structured questionnaire developed by adapting items from previously validated measures reported in the literature. The instrument comprised 45-item construct-specific measures. Sports Participation (SP), Educational Attainment (EA), Health-Related Behaviors (HRB), and Physical Health Outcomes (PHO) were each assessed using five items. Sports Participation covered frequency and duration of participation, type of sports involvement, level of engagement, and commitment to sporting activities. Educational Attainment covered educational level and status, perceived academic progress, and education-related developmental characteristics. Health-Related Behaviors assessed routine physical activity, healthy dietary practices, adequate sleep and recovery, sedentary habits, avoidance of harmful behaviors, and awareness of preventive health practices. Physical Health Outcomes covered perceived physical fitness, body-weight status, energy level, general health condition, illness frequency, and ability to perform daily physical activities. Each construct was treated as a single overall measure, and no separate subscales were used. Composite scores were generated by calculating the average of the five items representing each construct, resulting in mean values between 1.00 and 5.00. Higher scores reflected stronger levels of the corresponding construct, while higher PHO scores represented improved perceived physical health. For descriptive interpretation, the mean scores were grouped into the following categories: 1.00–1.80 as very low, 1.81–2.60 as low, 2.61–3.40 as moderate, 3.41–4.20 as high, and 4.21–5.00 as very high. These classifications were applied only for descriptive interpretation and were not considered standardized thresholds or diagnostic criteria.

### Assessment of instrument validity and reliability

The questionnaire’s content validity and contextual suitability were assessed through expert review. The panel of experts was composed of three specialists in the fields of sports sciences, public health and educational research. The items of the questionnaire were evaluated by experts for relevance, clarity, comprehensibility and consistency with the constructs of the study. The feedback led to minor changes in the wording of items and removal of ambiguity. A pilot test was conducted with 30 adults aged 18 years or above who had previously participated in organized or informal sporting activities and characteristic similar to those of the target study population. The pilot sample comprised individuals recruited from educational institutions, community settings and recreational or sports-related backgrounds. They scored the items of the questionnaire in terms of clarity, understanding, quality of wording and appropriateness overall. Minor changes to the item wording were made before the main data collection phase based on feedback from the pilot study.

Internal reliability was assessed using Cronbach’s alpha coefficients together with corrected item total correlations. Cronbach’s alpha values across the four constructs ranged from 0.82 to 0.88, which is above the recommended reliability criterion of 0.70 (43). The corrected item total correlation values varied between 0.604 and 0.746, indicating that all items had met the minimum standard of 0.30.

Suitability of the dataset for exploratory factor analysis was assessed through the Kaiser-Meyer-Olkin (KMO) measure and Bartlett’s test of sphericity. The Kaiser-Meyer-Olkin (KMO) value was 0.842 and Bartlett’s test was significant, *χ*^2^ (190) = 2846.317, *p* < 0.001, demonstrating that the correlation matrix was appropriate for factor analysis. The exploratory factor analysis produced four factors that were in line with the proposed constructs. The factor loading values ranged from 0.681 to 0.872. The four factors accounted for 70.58% of the total variance. Results section contains comprehensive findings regarding reliability and validity.

### Data collection procedure

Ethical clearance for the study was granted by the Research Ethics Committee of Xinyang Normal University, China (Approval no. 2025–033) before data collection was initiated. All study procedures were conducted in accordance with the ethical principles outlined in the Declaration of Helsinki. The study was conducted from October 2025 to December 2025. Participant recruitment and questionnaire administration were carried out during this period through educational institutions, community networks, sports clubs, and online platforms.

Before taking part in the study the subjects were informed about the study objectives, the voluntary nature of the participation and the confidential handling of their responses. Informed consent was obtained and questionnaires were completed. Questionnaire items were clearly and understandably worded, confidentiality was emphasised, and the order of items was arranged to minimise systematic response patterns, reducing response and common method biases (44).

### Statistical analysis

Data were analysed using IBM SPSS Statistics version 32 and the PROCESS macro version 5.0 for mediation analysis. First, data screening was conducted to identify responses that were incomplete, duplicate, inconsistent and otherwise unusable. For the four study constructs (Sports Participation, Educational Attainment, Health-Related Behaviors and Physical Health Outcomes), composite scores were created from the average of the values of the items used to represent each construct.

The psychometric properties of the measuring tool were evaluated through Cronbach’s alpha, corrected item–total correlations, the Kaiser-Meyer-Olkin (KMO) measure of sampling adequacy, Bartlett’s test of sphericity, and exploratory factor analysis (EFA). Descriptive statistics were used to compute means and standard deviations for the main study variables. A Pearson correlation analysis was then conducted to assess the strength and direction of the pairwise relationships between Sports Participation, Educational Attainment, Health-Related Behaviors and Physical Health Outcomes as the main study variables.

Pearson correlation analysis was used because the variables in the study were operationalised as composite scores calculated from multiple questionnaire items and therefore were considered to be approximately continuous measures. In the original analysis, formal tests for normality were not conducted, but the analytic sample size was relatively large (N = 520), which reduces the sensitivity of parametric estimates to moderate departures from normality. Pearson correlation is generally considered fairly robust to moderate non-normality in large samples when relationships are roughly linear and not dominated by extreme observations. Homogeneity of variance was not a prerequisite for Pearson correlation, as the analysis was concerned with associations between continuous composite variables rather than comparisons of means between independent groups. Therefore, the magnitude and direction of bivariate relationships between the constructs of the study were determined using the Pearson correlation.

Ordinary least-squares (OLS) regression was used to test the direct predictive relationships specified in the conceptual framework. Simple linear regression models were used to estimate pathways from sports participation to educational attainment and health-related behaviors and multiple linear regression models were used to estimate Physical Health Outcomes from Sports Participation, Educational Attainment and Health-Related Behaviors simultaneously. The total contribution of predictor variables to Physical Health Outcomes was calculated using the full regression model. Parallel mediation analysis was conducted to determine if Educational Attainment and Health-Related Behaviors were mediating variables in the relationship between Sports Participation and Physical Health Outcomes. Indirect effects were estimated with 5,000 bootstrap resamples and 95% confidence intervals. A mediation effect was statistically significant when the bootstrap confidence interval for the mediation effect did not contain zero. Statistical significance for all other inferential analyses was set at *p* < 0.05. The statistical analysis was not done with formal normality tests, and therefore we do not claim any further results of normality-tests. The Pearson correlation was chosen due to the large sample size, the use of multi-item composite measures and because this method tolerates moderate deviations from normality. No specific prerequisite of homogeneity of variance was required for the correlation analysis as no within-group mean comparison was performed.

## Results

The findings are presented according to the structure and sequence of the proposed research model. Initially, participant demographic characteristics are reported, followed by analyses of measurement reliability and validity, descriptive statistics, correlation results, direct and indirect effects from the parallel mediation model, and the final overall model findings.

The final analysis comprised 520 adults aged 18 years or above who had participated in either organized or informal sporting activities. Table 1 shows that the sample was relatively balanced gender-wise (52% males and 48% females). Regarding educational status, 22% completed secondary education, 58% were enrolled or completed undergraduate education and 20% had postgraduate education. Regarding residential background, 61% of the participants were from urban areas and 39% from rural areas. The sample demographic composition indicates that it was representative of participants with different educational and residential backgrounds.

**Table 1:** Demographic characteristics of participants (*N* = 520).

Demographic variable	Category	Percentage (%)
Gender	Male	52
	Female	48
Educational status	Secondary education	22
	Undergraduate	58
	Postgraduate	20
Residential background	Urban	61
	Rural	39

N, Number of participants.

### Measurement reliability and validity

As presented in Table 2, Cronbach’s alpha values across the four constructs varied between 0.82 and 0.88, demonstrating satisfactory internal consistency.

**Table 2:** Results of the reliability assessment.

Construct	Number of items	Cronbach’s alpha	Interpretation
Sports participation (SP)	5	0.86	Good reliability
Educational attainment (EA)	5	0.82	Good reliability
Health-related behaviors (HRB)	5	0.88	Good reliability
Physical health outcomes (PHO)	5	0.85	Good reliability

SP, Sports Participation; EA, Educational Attainment; HRB, Health-Related Behaviors; PHO, Physical Health Outcomes.

The KMO value was 0.842 and the Bartlett’s test of sphericity was significant [*χ*^2^ (190) = 2846.317, *p* < 0.001] as shown in Table 3, indicating that the data set was appropriate for factor analysis.

**Table 3:** Results of the KMO and Bartlett’s tests.

Test	Value	Interpretation
Kaiser-Meyer-Olkin sampling adequacy measure	0.842	Suitable for factor analysis
Bartlett’s test of sphericity	2846.317	Significant
Degree of freedom	190	—
Significance level	0.000	Appropriate for factor analysis

KMO denotes the Kaiser-Meyer-Olkin measure of sampling adequacy; *χ*^2^ denotes the chi-square statistic; df represents degrees of freedom; and *p* represents the probability value.

As shown in Table 4, corrected item total correlations varied from 0.604 to 0.746. Every item exceeded the minimum acceptance criterion and was therefore retained for further analysis.

**Table 4:** Corrected item–total correlation analysis.

Construct	Item	Corrected item-total correlation	Interpretation
Sports participation	SP1	0.714	Acceptable
	SP2	0.682	Acceptable
	SP3	0.701	Acceptable
	SP4	0.653	Acceptable
	SP5	0.621	Acceptable
Educational attainment	EA1	0.667	Acceptable
	EA2	0.642	Acceptable
	EA3	0.619	Acceptable
	EA4	0.604	Acceptable
	EA5	0.631	Acceptable
Health-related behaviors	HRB1	0.746	Acceptable
	HRB2	0.722	Acceptable
	HRB3	0.695	Acceptable
	HRB4	0.671	Acceptable
	HRB5	0.648	Acceptable
Physical health outcomes	PHO1	0.718	Acceptable
	PHO2	0.690	Acceptable
	PHO3	0.664	Acceptable
	PHO4	0.638	Acceptable
	PHO5	0.612	Acceptable

SP, Sports Participation; EA, Educational Attainment; HRB, Health-Related Behaviors; PHO, Physical Health Outcomes. SP1–SP5, EA1–EA5, HRB1–HRB5 and PHO1–PHO5 are the respective measurement items of each construct.

The factor loading values ranged from 0.681 to 0.872 (see Table 5), which suggested that all measurement items had acceptable loading levels on their respective constructs.

**Table 5:** Results of exploratory factor analysis and factor loadings.

Construct	Item	Factor loading
Sports participation	SP1	0.842
Sports participation	SP2	0.816
Sports participation	SP3	0.798
Sports participation	SP4	0.735
Sports participation	SP5	0.681
Educational attainment	EA1	0.811
Educational attainment	EA2	0.786
Educational attainment	EA3	0.749
Educational attainment	EA4	0.704
Educational attainment	EA5	0.692
Health-related behaviors	HRB1	0.872
Health-related behaviors	HRB2	0.845
Health-related behaviors	HRB3	0.803
Health-related behaviors	HRB4	0.758
Health-related behaviors	HRB5	0.724
Physical health outcomes	PHO1	0.851
Physical health outcomes	PHO2	0.824
Physical health outcomes	PHO3	0.789
Physical health outcomes	PHO4	0.742
Physical health outcomes	PHO5	0.705

SP refers to Sports Participation; EA represents Educational Attainment; HRB indicates Health-Related Behaviors; PHO denotes Physical Health Outcomes; and EFA refers to Exploratory Factor Analysis.

As indicated in Table 6, the four extracted factors explained a total variance of 70.58%. Thus, the four-factor model identified explained a significant proportion of the variability among the measurement items.

**Table 6:** Explained total variance.

Factor	Construct	Eigenvalue	Variance explained (%)	Cumulative variance (%)
1	Health-related behaviors	4.218	21.09	21.09
2	Sports participation	3.746	18.73	39.82
3	Physical health outcomes	3.312	16.56	56.38
4	Educational attainment	2.841	14.20	70.58

% = percentage of variance explained.

### Descriptive statistics and correlations

As reported in Table 7, Health-Related Behaviors yielded the highest average score (*M* = 4.15, SD = 0.70), while Educational Attainment showed the lowest average score (*M* = 4.05, SD = 0.68). All variables showed mean scores greater than 4.00 across the study.

**Table 7:** Summary descriptive statistics.

Variable	*N*	Mean	Standard Deviation
Sports participation	520	4.10	0.72
Educational attainment	520	4.05	0.68
Health-related behaviors	520	4.15	0.70
Physical health outcomes	520	4.12	0.74

*N* denotes the number of participants; M represents the mean; SD denotes the standard deviation.

Table 8 shows that all the study variables were positively and significantly correlated with each other (*p* < 0.001). The strongest correlation was found between Health-Related Behaviors and Physical Health Outcomes (*r* = 0.64), followed by Sports Participation and Physical Health Outcomes (*r* = 0.63). Table 8 shows that all study variables were positively and significantly correlated with each other (*p* < 0.001). Sports Participation was also positively associated with Health-Related Behaviors (*r* = 0.60) and Educational Attainment (*r* = 0.55).

**Table 8:** Pearson correlation analysis.

Variable	Sports participation	Educational attainment	Health-related behaviors	Physical health outcomes
Sports participation	1.00			
Educational attainment	0.55***	1.00		
Health-related behaviors	0.60***	0.52***	1.00	
Physical health outcomes	0.63***	0.59***	0.64***	1.00

SP, Sports Participation; EA, Educational Attainment; HRB, Health Related Behaviors; PHO, Physical Health Outcomes; *r*, Pearson correlation coefficient; ***p < 0.001.

As presented in Table 9, all estimated regression pathways were positive and statistically significant. Sports Participation emerged as a significant predictor of Physical Health Outcomes [*β* = 0.410, SE = 0.038, *t* = 10.79, *p* < 0.001, 95% CI (0.336, 0.484)]. Educational Attainment [*β* = 0.550, SE = 0.037, *t* = 14.86, *p* < 0.001, 95% CI (0.477, 0.623)] and Health-Related Behaviors [*β* = 0.600, SE = 0.035, *t* = 17.14, *p* < 0.001, 95% CI (0.531, 0.669)] were likewise significant predictors.

**Table 9:** Regression coefficients for paths in the parallel mediation model.

Path	Relationship	β	SE	t	p	95% CI	Result
H1	Sports participation → physical health outcomes	0.410	0.038	10.79	<0.001	[0.336, 0.484]	Supported
H2 (a₁)	Sports participation → educational attainment	0.550	0.037	14.86	<0.001	[0.477, 0.623]	Supported
a₂	Sports participation → health-related behaviors	0.600	0.035	17.14	<0.001	[0.531, 0.669]	Significant
H3 (b₁)	Educational attainment → physical health outcomes	0.360	0.036	10.00	<0.001	[0.289, 0.431]	Supported
b₂	Health-related behaviors → physical health outcomes	0.373	0.037	10.08	<0.001	[0.300, 0.446]	Significant

SP, Sports Participation; EA, Educational Attainment; HRB, Health-related Behaviors; PHO, Physical Health Outcomes; *β*, Standardised Regression Coefficient; SE, Standard Error; *t*, *t* Statistic; *p*, Probability Value; CI, Confidence Interval; LLCI, Lower Limit of the 95% Confidence Interval; ULCI, Upper Limit of the 95% Confidence Interval. In the parallel mediation model, SP was the predictor, PHO was the outcome, and EA and HRB were the parallel mediators. Statistical significance was set at *p* value < 0.05.

### Parallel mediation analysis

As presented in Table 10, Sports Participation demonstrated a statistically significant overall effect on Physical Health Outcomes [effect = 0.742, SE = 0.044, 95% CI (0.656, 0.828)]. After accounting for the two parallel mediators, the direct effect continued to reach statistical significance [effect = 0.320, SE = 0.051, 95% CI (0.220, 0.420)]. Statistically significant indirect pathways were identified through Educational Attainment [indirect effect = 0.198, SE = 0.035, 95% CI (0.132, 0.271)] and Health-Related Behaviors [indirect effect = 0.224, SE = 0.040, 95% CI (0.151, 0.306)]. The combined indirect effect was estimated at 0.422 [SE = 0.053, 95% CI (0.322, 0.527)], indicating that both Educational Attainment and Health-Related Behaviors acted as significant mediators of the relationship linking Sports Participation with Physical Health Outcomes.

**Table 10:** Parallel mediation analysis.

Effect	Effect estimate	SE	95% LLCI	95% ULCI	Result
Total effect: SP → PHO	0.742	0.044	0.656	0.828	Significant
Direct effect: SP → PHO	0.320	0.051	0.220	0.420	Significant
Indirect effect via EA	0.198	0.035	0.132	0.271	Supported
Indirect effect via HRB	0.224	0.040	0.151	0.306	Supported
Combined indirect effect	0.422	0.053	0.322	0.527	Significant

SP, Sports Participation; EA, Educational Attainment; HRB, Health-Related Behaviors; PHO, Physical Health Outcomes; SE denotes the standard error; LLCI, lower limit of the 95% confidence interval; ULCI, upper limit of the 95% confidence interval. EA and HRB were simultaneously specified as parallel mediators, and the indirect effects were estimated using 5,000 bootstrap samples, with significance inferred when the corresponding 95% bootstrap confidence interval excluded zero.

Figure 2 presents the parallel mediation model with the estimated path coefficients. Sports Participation was a significant predictor of Educational Attainment (*β* = 0.550, *p* < 0.001) and Health-Related Behaviors (*β* = 0.600, *p* < 0.001). In turn, Educational Attainment (*β* = 0.360, *p* < 0.001) and Health-Related Behaviors (*β* = 0.373, *p* < 0.001) also emerged as a significant predictor of Physical Health Outcomes. The direct association between Sports Participation and Physical Health Outcomes continued to be statistically significant after the inclusion of the two mediators [effect = 0.320, SE = 0.051, 95% CI (0.220, 0.420)], indicating partial mediation. Statistically significant indirect effects were identified through Educational Attainment [effect = 0.198, 95% CI (0.132, 0.271)] and Health-Related Behaviors [effect = 0.224, 95% CI (0.151, 0.306)], while the combined indirect effect was 0.422 [95% CI (0.322, 0.527)]. These findings visually and statistically support the proposed parallel mediation model.

**Figure 2:**
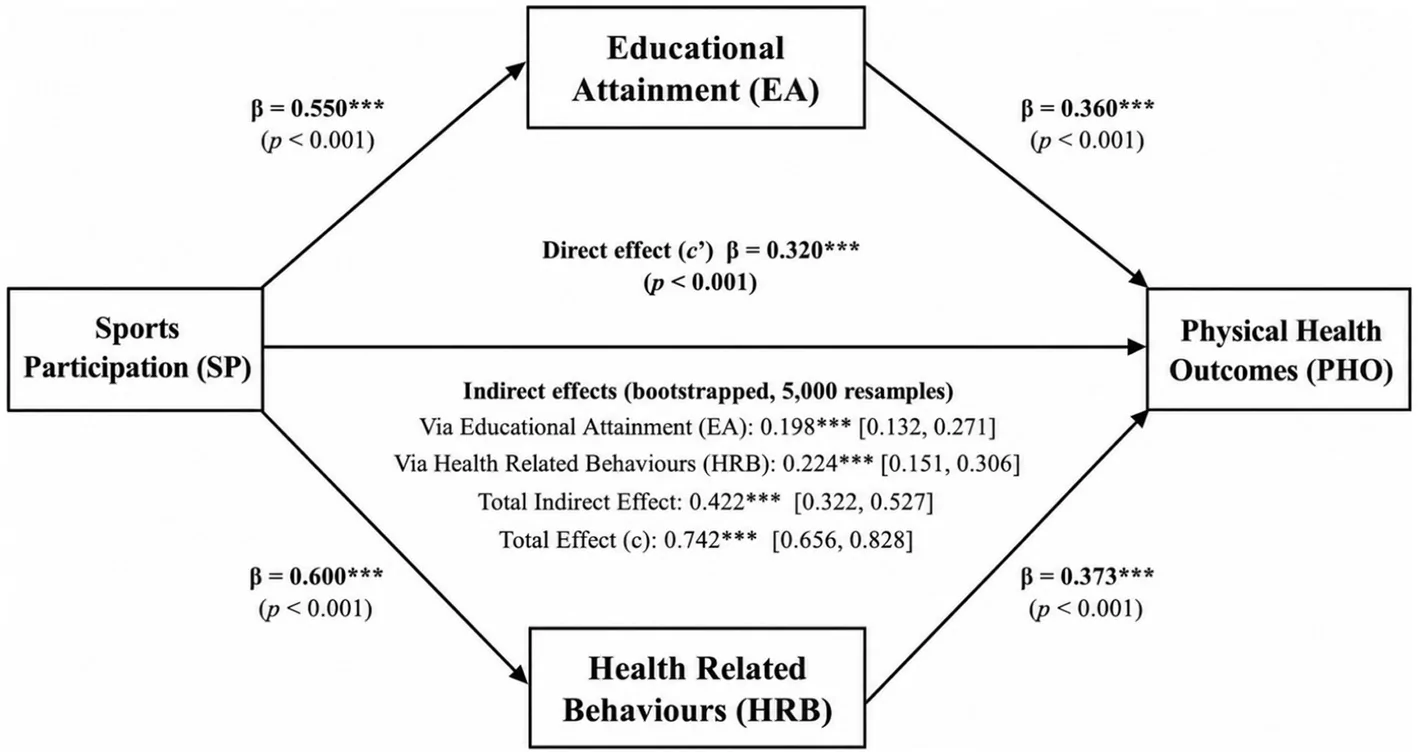
Parallel mediation model with estimated path coefficients.

### Model summary

As shown in Table 11, Sports Participation, Educational Attainment, and Health-Related Behaviors explained 58% of the variance in Physical Health Outcomes (*R*^2^ = 0.58, adjusted *R*^2^ = 0.57). The overall regression model was statistically significant, *F* (3, 516) = 236.84, *p* < 0.001, and the RMSE was 0.481.

**Table 11:** Model summary for physical health outcomes.

Dependent variable	Predictors	*N*	*R* ^2^	Adjusted *R*^2^	*F*	df1	df2	*p*	RMSE
Physical health outcomes	Sports participation, educational attainment, health-related behaviors	520	0.58	0.57	236.84	3	516	<0.001	0.481

*N*, Sample size; *R*^2^ denotes the coefficient of determination; Adjusted *R*^2^ = adjusted coefficient of determination; F, F statistic; df1, numerator degrees of freedom; df2, denominator degrees of freedom; *p*, probability value; RMSE, represents the root mean square error.

## Discussion

The study sample comprised 520 adults aged 18 years and over who took part in either organized or informal sport. The sample was fairly evenly split by gender (52% male, 48% female) and included people from a broad spectrum of educational and residential backgrounds. The positive association between the participation in sports and physical health observed is consistent with Song and Shi (45), although their population was substantially different from the present sample. Song and Shi (45) analysed data from 42,777 children and adolescents in the United States (mean age = 9.4 years; 48.3% girls) and found that participants who participated in sports were more likely to report better overall health than those who did not participate in sports [OR = 1.92, 95% CI (1.83, 2.02)]. Thus, despite the differences in age and sample size, both studies suggest a positive association between sports participation and perceived health outcomes.

The same pattern can be observed for the educational pathway. Chen et al. (46) studied 6,946 U. S. high-school students aged 14–17 years and found that adolescents who were involved in one, two or at least three sports teams had increasingly higher odds of better academic performance than those who were not involved. Similarly, Muñoz-Bullón et al. (15) used longitudinal panel data from undergraduate students at a Spanish university between 2008 and 2014 and found that participation in formal sporting activities was associated with higher academic achievement. The present results are generally consistent with the positive association between sports and education found in the adult sample. However, not all the previous evidence is consistent. Using data from the National Longitudinal Study of Adolescent Health, a survey of U. S. high-school students, Rees and Sabia (47) demonstrated that the evidence for the notion that sports participation independently enhances academic performance is limited. When taking into account unobserved individual characteristics, the estimated effects were substantially attenuated or even reversed (48). This difference raises the possibility that heterogeneity in participant age, educational context, measurement methods and analytical strategies could help to explain the differences seen across studies, and further underlines that the associations identified in this cross-sectional analysis should be interpreted as predictive associations and not as evidence of causal effects (53).

This interconnected pattern may be interpreted within a social-ecological framework (54). The Social Ecological Model suggests that health outcomes arise from interactions between individual behaviors and broader interpersonal, organisational, community, and policy-level influences (49). In this context, participation in sport is not an isolated individual behavior. It is often found in schools, universities, clubs, communities and other social environments that can provide routine, social interaction, institutional engagement and learning opportunities. These contexts potentially influence physical activity behaviors, education, and everyday health behaviors. Accordingly, the proposed model indicates that the association between sports participation and health may operate through interconnected social and behavioral mechanisms independent of a single physiology.

In this instance, educational attainment is a relatively distal social-cognitive pathway (50). Sport participation may be associated with traits such as discipline, persistence, time management, goal orientation, teamwork and engagement with institutions, which may also support progress in educational development (51). Education, in turn, is a well-known social determinant of health, as it is related to health literacy, socioeconomic opportunities, access to resources, living and employment conditions, together with the capacity to make well-informed decisions about health (52). The mediating role of educational attainment may thus point to a pathway from developmental and institutional experiences related to sport to social and cognitive resources relevant for health (53). This pathway should not be understood unidirectional as well. Access to sporting opportunities may also be facilitated by educational resources and socioeconomic advantage, indicating potential reciprocal relationships between education and participation (16).

Within this model health-related behaviors are a relatively proximal behavioral mechanism (54). Regular engagement in sports may be associated with lifestyle routines related to physical activity, nutrition, sleep, recovery, sedentary habits, and avoidance of harmful behaviors (55). Earlier research has similarly identified links between engagement in physical activity and healthier lifestyle practices (56). These behaviors may represent a more direct pathway through which sports involvement contributes to physical well-being. The mediation through health-related behaviors therefore suggests that the health relevance of sport may extend beyond the physical activity performed beyond the act of participation itself toward the broader behavioral patterns that develop alongside sustained involvement (57).

Taken together, the two mediating pathways provide complementary explanations. Educational attainment reflects broader social, cognitive, and resource-related conditions, whereas health-related behaviors represent everyday behavioral practices more directly connected with health. Their simultaneous role is consistent with a Social Determinants of Health perspective, according to which individual health is shaped by both personal behavior and access to social and educational resources (58). These findings extend existing literature by integrating these pathways into one comprehensive framework instead of examining sport–health, sport–education, and education–health associations as separate relationships.

Several methodological strengths enhance the value of the present study. First, the analysis was based on a relatively large sample of 520 adults with variation in gender, education status and residential background, allowing the proposed relationships to be examined across a heterogeneous participant group. Secondly, this study considered the four constructs of sports participation, educational attainment, health-related behaviors, and physical health outcomes simultaneously in one integrated framework rather than independent relationships. Third, Cronbach’s alpha, corrected item–total correlation analysis, the Kaiser–Meyer–Olkin index, Bartlett’s sphericity test, and exploratory factor analysis were used to assess the reliability and construct validity of the measurement instrument. The results showed satisfactory psychometric performance for all four constructs. Finally, the use of regression analysis and parallel mediation analysis using 5,000 bootstrap samples together with 95% confidence intervals provided a more robust examination of the proposed indirect pathways than simple bivariate relationships alone. These methodologic features, taken together, strengthen the methodological consistency and analytical rigor of the study, while acknowledging the limitations of cross-sectional and convenience-sampling design.

These findings also have practical implications of the integrated model. Programs aimed at promoting participation should not be exclusively focused on increasing physical activity. Sporting opportunities, when integrated into supportive settings that foster health literacy, healthy eating, adequate sleep, decreased sedentary behavior, and continued educational involvement, can provide increased public health value for educational institutions and community organisations. Another important point is accessibility, since socioeconomic and environmental factors can impact people’s ability to participate in organized sports (59). Coordinated strategies that blend accessible sporting opportunities with health education and supportive institutional environments may therefore be of benefit to schools, universities, community organisations and public health agencies. Given the cross-sectional design of the study, the significant indirect associations should be viewed as mediation effects within the proposed analytical model rather than as evidence of temporal causation. The results do not indicate that participation leads to changes in educational outcomes or health-related behaviors. However, the identified parallel mediation pattern provides a useful foundation for future longitudinal research that explores how educational resources, behavioral practices, and sporting contexts affect physical health over time.

### Study limitations and directions for future research

There are several limitations to consider when considering the study findings. First, the cross-sectional design precludes conclusions regarding the temporal ordering or causal relations among study variables. While the proposed mediation framework points to directional associations, longitudinal studies are required to assess whether participation precedes changes in educational development, health-related behaviors, and physical health across time.

Second, the study relied on self-reported questionnaire responses, which may be affected by recall bias, socially desirable responding, and common-method variance. Future studies could also supplement self-reported measures with objective measures such as physical fitness assessments, body composition measurements, medical indicators, or wearable device data.

Using convenience sampling together with stratified outreach procedures may further restrict the extent to which these findings can be generalized. Future research should incorporate a broader range of individuals from different geographic regions, socioeconomic statuses, educational backgrounds, and age brackets. Special attention should be paid to socioeconomic status, access to sporting facilities, family and social support, health literacy and institutional environments, as these may affect opportunities to participate and health-related outcomes. Longitudinal and mixed-method research could further clarify the temporal ordering and contextual mechanisms underlying the relationships identified in the present model.

## Conclusion

This study adds to the sports and public health literature by demonstrating that the association between sports participation and physical health may operate through interconnected physical, educational, and behavioral mechanisms. The main contribution is not only that more participation is associated with better health but that education and health behaviors are complementary mechanisms in this relationship. Education is a broader track in terms of social and resources. Health behaviors are the daily practices that can enhance health benefits.

Therefore, the research supports a more integrated understanding of sport as part of a wider public health environment, and not as an isolated form of physical activity. Hence, approaches combining accessible sporting opportunities with educational support, health literacy and healthier daily habits may be of greater value than interventions solely based on participation. Although longitudinal research is required to examine the temporal sequence of these relationships, the proposed framework provides a basis for future inquiry as to how social, educational, and behavioral factors jointly influence the relationship linking sports participation with physical health.

## Data Availability

The original contributions presented in the study are included in the article/supplementary material, further inquiries can be directed to the corresponding author/s.
